# Photocatalytic Properties of *g*-C_3_N_4_–TiO_2_ Heterojunctions under UV and Visible Light Conditions

**DOI:** 10.3390/ma9040286

**Published:** 2016-04-14

**Authors:** Rachel Fagan, Declan E. McCormack, Steven J. Hinder, Suresh C. Pillai

**Affiliations:** 1 Centre for Research in Engineering Surface Technology (CREST), FOCAS Institute, Dublin Institute of Technology, Kevin St, Dublin 8, Ireland; rachel.fagan@mydit.ie; 2 School of Chemical and Pharmaceutical Sciences, Dublin Institute of Technology, Kevin St., Dublin 8, Ireland; 3 The Surface Analysis Laboratory, Department of Mechanical Engineering Sciences, University of Surrey, Guildford, Surrey GU2 7XH, UK; s.hinder@surrey.ac.uk; 4 Nanotechnology Research Group, Department of Environmental Sciences, Institute of Technology Sligo, Sligo, Ireland; 5 Centre for Precision Engineering, Materials and Manufacturing Research (PEM), Institute of Technology Sligo, Sligo, Ireland

**Keywords:** titanium dioxide, graphitic carbon nitride, photocatalytic activity

## Abstract

Graphitic carbon nitride (*g*-C_3_N_4_) and titanium dioxide (TiO_2_) were chosen as a model system to investigate photocatalytic abilities of heterojunction system under UV and visible light conditions. The use of *g*-C_3_N_4_ has been shown to be effective in the reduction in recombination through the interaction between the two interfaces of TiO_2_ and *g*-C_3_N_4_. A simple method of preparing *g*-C_3_N_4_ through the pyrolysis of melamine was employed, which was then added to undoped TiO_2_ material to form the *g*-C_3_N_4_–TiO_2_ system. These materials were then fully characterized by X-ray diffraction (XRD), Brunauer Emmett Teller (BET), and various spectroscopic techniques including Raman, X-ray photoelectron spectroscopy (XPS), Fourier transform infrared spectroscopy (FT-IR), diffuse absorbance, and photoluminescence analysis. Photocatalysis studies were conducted using the model dye, rhodamine 6G utilizing visible and UV light irradiation. Raman spectroscopy confirmed that a composite of the materials was formed as opposed to a mixture of the two. Using XPS analysis, a shift in the nitrogen peak to that indicative of substitutional nitrogen was detected for all doped samples. This is then mirrored in the diffuse absorbance results, which show a clear decrease in band gap values for these samples, showing the effective band gap alteration achieved through this preparation process. When *g*-C_3_N_4_–TiO_2_ samples were analyzed under visible light irradiation, no significant improvement was observed compared that of pure TiO_2_. However, under UV light irradiation conditions, the photocatalytic ability of the doped samples exhibited an increased reactivity when compared to the undoped TiO_2_ (0.130 min^−1^), with 4% *g*-C_3_N_4_–TiO_2_ (0.187 min^−1^), showing a 43.9% increase in reactivity. Further doping to 8% *g*-C_3_N_4_–TiO_2_ lead to a decrease in reactivity against rhodamine 6G. BET analysis determined that the surface area of the 4% and 8% *g*-C_3_N_4_–TiO_2_ samples were very similar, with values of 29.4 and 28.5 m^2^/g, respectively, suggesting that the actual surface area is not a contributing factor. This could be due to an overloading of the system with covering of the active sites resulting in a lower reaction rate. XPS analysis showed that surface hydroxyl radicals and oxygen vacancies are not being formed throughout this preparation. Therefore, it can be suggested that the increased photocatalytic reaction rates are due to successful interfacial interactions with the *g*-C_3_N_4_-doped TiO_2_ systems.

## 1. Introduction

Heterojunction photocatalyst systems are deemed to be an excellent option to improve the photocatalytic behavior of a material. These heterojunction systems exhibit improved charge separations and increased lifetimes of the charge carriers [1]. Prime examples include that of the anatase–rutile [2] or the anatase–brookite heterojunction system [3], which both promote the effective transfer of photo-excited electrons and favor electron-hole separation. Graphitic carbon nitride (*g*-C_3_N_4_) has been shown to be efficient at producing hydrogen through the oxidation of organic species [4,5]. Therefore, a *g*-C_3_N_4_-doped titanium dioxide (TiO_2_) system was chosen to be studied as a continuation of this study.

Graphitic carbon nitride (*g*-C_3_N_4_), an allotrope of carbon nitride, is one material of interest due its being regarded as having the best stability under ambient conditions. This semiconductor material has a measured band gap value of 2.7 eV consistent with an optical wavelength of 460 nm, thus making the material slightly yellow in color and active in the visible light region. With a medium band gap as well as thermal and chemical stability in ambient environment, it becomes one of the most promising photocatalytic materials [6]. Several publications in recent years have highlighted the effectiveness of *g*-C_3_N_4_ as a photocatalyst [7,8,9,10].

Several publications in recent years have highlighted the effectiveness of *g*-C_3_N_4_ as a photocatalyst in areas such as the selective oxidation of alcohols and hydrocarbons [4,11], and as a good photocatalytic performer in relation to hydrogen or oxygen production via water splitting with the use of visible light irradiation [5]. It has strong reduction reaction properties owing to the high potential of the conduction band but inferior oxidation capabilities due to its valence band located at about 1.4 eV *vs.* NHE, resulting in a small thermodynamic driving force for water or organic pollutants oxidation. To address the inferior oxidation related issues of *g*-C_3_N_4_, various composites have been prepared in recent years to enhance its activities. The effectiveness of *g*-C_3_N_4_ as a dopant of TiO_2_ for the enhancement of photocatalytic activities due to a reduction in the recombination rate [7,9,10,12,13,14,15,16], with nitrogen-doped TiO_2_ [17] surface fluorinated TiO_2_ [18] and S-TiO_2_ [19] also prepared and studied.

Several groups have studied this material as composite systems with WO_3_ [20,21,22] and multi-walled nanotubes (MWNTs) [23], which were both proven to promote efficient charge separation through interfacial interaction. A composite system with SiO_2_ [24] showed an increased surface area and subsequently an improved photocatalytic degradation rate. Another system studied was the incorporation of metal nanoparticles such as Ag [25], proven to facilitate charge separation. A system including Ag@AgBr [26] was deemed effective in enhancing the photocatalytic rate of reaction through the formation of a Z-scheme reaction, which keeps the *e*_CB_^−^ in the conduction band (CB) of *g*-C_3_N_4_ with a high reduction capability and *h*_VB_^+^ with high oxidation capability in the valence band (VB) of AgBr. Other systems studied were Fe-doped *g*-C_3_N_4_ [27], a combination of Fe and phosphorus [28], which was identified as improving the photocatalytic activity of the system through the retardation of the crystal growth, enhancement of the surface area, decreased band gap energy, and increased separation of electron-hole pairs. Successful interfacial interaction and charge separation was achieved upon the addition of C_60_ [29], which exhibited a significant enhancement on the photocatalytic performance. This is due to a better separation of the photo-induced electron-hole pairs and longer lifetime of the photo-generated charge carriers of the bulk *g*-C_3_N_4_. When tested against phenol and methylene blue dye, this effect promotes an improved photocatalytic activity of the *g*-C_3_N_4_. [30]. Another composite is one of tungsten (VI) oxide and *g*-C_3_N_4_ (WO_3_–*g*-C_3_N_4_) [22] with the WO_3_ used with the intention of providing a combination partner for the *g*-C_3_N_4_, as it is well known as an oxidation part photocatalyst for the Z-scheme photocatalytic water splitting, where Z-scheme involves a means for utilizing both high oxidation and reduction abilities using visible light irradiation [21,31]. CdS–*g*-C_3_N_4_ [32] is an effective photocatalyst used for the production of aldehydes from the oxidation of aromatic alcohols and the conversion of nitrobenzene to aniline through a reduction reaction. This is achieved through direct hole oxidation and direct electron reduction, respectively.

Throughout the literature, several methods for the production of *g*-C_3_N_4_ have been outlined. Generally, these methods have either included the heat treatment of thiourea [33], urea [17], or, most often, an organic compound melamine [7,13,20,24,25,32,34,35,36,37,38]. Melamine (C_3_H_6_N_6_) has uses mainly in the production of plastics, insulation, soundproofing, and cleaning products and can also act as a flame retardant when mixed with certain resins. Melamine also provides carbon and nitrogen for the doping of TiO_2_ and is converted to *g*-C_3_N_4_ upon heat treatment, with Teter and Hemley describing the resulting *g*-C_3_N_4_ as the perfect de-ammonation polycondensate of melamine [39]. Graphitic carbon nitride is produced through the pyrolysis of melamine, which is then converted to the melam structure before further conversion to *g*-C_3_N_4_ by thermal condensation [9].

The use of melamine to prepare *g*-C_3_N_4_ is studied with the prepared *g*-C_3_N_4_ material mixed with undoped TiO_2_ to effectively achieve an interfacial interaction between the two components with the aim of obtaining an improved photocatalyst. This study aims to determine the effectiveness of *g*-C_3_N_4_ and TiO_2_ heterojunctions, and its potential influence on the modifications of the band structure. The efficiency of any potential carbon doping was measured by a combination of X-ray photoelectron spectroscopy (XPS), X-ray diffraction (XRD), and various spectroscopic measurements along with the analysis of its photocatalytic degradation of the target dye, rhodamine 6G, in an aqueous solution, under visible and UV light irradiation. It should be noted that the standard redox potential E^0^ (^−^OH–OH^−^, 1.99 V) is reported to be more positive than the valence band of *g*-C_3_N_4_ (1.65 V). Therefore, the holes generated in the valence band of *g*-C_3_N_4_ cannot oxidize water to form hydroxyl radicals. Therefore, the holes are not found to be the major species for photocatalytic action. In addition, the holes possess a low oxidation potential (1.4 eV), which is not sufficient to generate oxidizing radicals for the degradation of organic dyes (e.g., rhodamine 6G). Based on these explanations the photocatalytic action can be attributed largely on the generation of reactive oxidation species induced by photo-generated electrons [40].

## 2. Experimental

### 2.1. Materials

In this study, titanium tetraisopropoxide (97.0%), isopropanol (99.0%), and melamine (99.0%) were purchased from Aldrich and were used without further purification to prepare the samples. The dye used for the photocatalytic study (rhodamine 6G) was obtained from Eastman and was of analytical reagent grade and used without further purification. Deionized water was used in all experiments.

### 2.2. Preparation of Nanomaterials

#### 2.2.1. Preparation of Titanium Dioxide

In a typical experiment to prepare titania, 37.44 mL (0.032 mol) of titanium isopropoxide (Ti(OPr)_4_) was added slowly with stirring to 329.6 mL (1.078 mol) of isopropanol. To this solution, a 480 mL (6.659 mol) of deionized water was added drop-wise. The precipitate was stirred for 2 h at room temperature (20 °C). The resulting solution was then irradiated in a CEM MARS 5 microwave system under ambient pressure at 400 W for 20 min. The irradiated precipitate was then filtered, and the solid obtained was washed with 100 mL of deionized water. The resulting solid was placed in an oven at 80 °C to dry.

#### 2.2.2. Preparation of *g*-C_3_N_4_

5 grams of melamine (C_3_H_6_N_6_) was weighed and ground for a period of 10 min. This finely ground powder was then calcined 500 °C with a ramp rate of 10 °C/min and held for a period of 2 h. An additional ramping step of 5 °C/min up to 520 °C, with a hold of 2 h, resulted in the production of *g*-C_3_N_4_.

#### 2.2.3. Preparation of *g*-C_3_N_4_–TiO_2_ Materials

*g*-C_3_N_4_–TiO_2_ was prepared by mixing the two components and grinding for a period of 10 min until a smooth, uniform powder was achieved. Samples were prepared in varying weight ratios of *g*-C_3_N_4_ to TiO_2_, *i.e.*, 2%, 4%, and 8% *g*-C_3_N_4_–TiO_2_. The resulting powder was calcined in air at 100 °C intervals ranging from 600 °C to 1000 °C with a ramp rate of 5 °C/min and held at these temperatures for a 2 h period.

### 2.3. Characterization

A combination of analytical techniques was used allowing the full characterization of the *g*-C_3_N_4_–TiO_2_ samples produced. These techniques included XRD using a Siemens D 500 X-ray diffractometer (Siemens, Germany) with the diffraction angles scanning from 2*θ* = 20°–80°, using a Cu K*α* radiation source. XRD was utilized in phase identification and the measurement of particle size and the anatase to rutile transition. The crystallite size for each sample was calculated using the Scherrer equation (Equation (1)) [41].

(1) $$D=\frac{0.9\lambda }{\beta cos\theta }$$

where *D* is the crystalline size, *λ* is the X-ray radiation wavelength (0.154 nm), *β* is the full line width at half-maximum height of the main intensity peak, and *θ* is Bragg’s angle.

XRD can also be used in order to calculate the percentage of anatase and rutile phases present in each sample analyzed. This is measured using the Spurr equation (Equation (2)) [42].

(2) $$\%\;rutile=\;\frac{1}{1+0.8[I_{A}(101)/I_{R}\left(110\right)]}$$

where *I*_A_ is the intensity of (101) anatase peak, and *I*_R_ is the intensity of (110) rutile peak.

Raman spectroscopy carried out using a Horiba Jobin Yvon LabRAM HR system was used to confirm the crystalline phase present in the desired samples. Samples were analyzed using the 50x objective lens at filter percentages between 1% and 100%, which varied according to the intensity recorded for each samples. A 300 grating and a scan range of 0–1200 cm^−1^ was used in all experiments, with each run having an exposure time of 3 s. The Spectrum GX-FTIR (Perkin Elmer, Waltham, MA, USA) spectrophotometer was used to confirm the formation of TiO_2_ and the molecular interactions achieved with the dopants present, measured over a range of 400–4000 cm^−1^ with an accumulation of 8 scans. In order to study the optical properties, the diffuse absorbance spectra of solid powder samples were measured employing an integrating sphere in absorbance mode. For this analysis, a Perkin Elmer Lambda 900 UV-Vis absorption spectrophotometer (Perkin Elmer, Waltham, MA, USA) was used, with scans running over a range of 300–600 nm. XPS analyses were performed on a Thermo Fisher Scientific Theta Probe spectrometer with an Al K*α* source (Thermo Fischer Scientific, Waltham, MA, USA). All samples were run in triplicate with a pass energy of 20 eV, and, due to the charging of the binding energy, samples were calibrated relative to the C 1s peak at 284 eV. A Perkin Elmer LS55B Luminescence Spectrometer was used for all photoluminescence analysis, conducted at room temperature (20 °C) with a Xe flash lamp pulsed at line frequency as the light source. Samples were prepared by mixing in KBr (1:20 sample/KBr) and then pressing to form a disc. An excitation wavelength of 350 nm with a 10 nm slit sufficiently excited the electrons and was measured over a scan range of 370–630 nm. BET surface area analyses were performed using a Gemini VII 2390 Surface Area Analyzer (Micrometrics, Norcross, GA, USA). The samples were degassed at 300 °C for 2 h, and the adsorption isotherms were obtained at −196.15 °C.

### 2.4. Photocatalytic Study

Photocatalysis studies were carried out with an aqueous solution of rhodamine 6G (4 mg/L) using UV light irradiation with two F15T8/BL lamps (EIKO, Shawnee, KS, USA) having an output of 15 W (350 nm). Visible light irradiation analysis was conducted with the use of two 840 Cool White, Spectra-Plus triphosphor lamps (Crompton Lamps, Bradford, UK) with power output of 15 W These studies were carried out to examine the effect of the TiO_2_ samples on the above dye. The results presented below are from using optimum conditions of dye concentration, sample concentration, and irradiation time.

25 mg of each sample was added to 50 mL of rhodamine 6G solution. The above suspension was stirred for 30 min in the dark to equilibrate and eliminate any error due to the initial adsorption effect. Aliquots were taken at 5-min intervals for a period of 30 min and then centrifuged to remove all solids remaining in the solution to avoid any error due to scattering. These aliquots were then analyzed using a UV-Vis spectrometer to measure their degradation properties. The photocatalytic reaction was assumed to obey pseudo-first-order kinetics, and the rate constant for degradation, *k*, was calculated from the first-order rate plot. Irradiation of rhodamine 6G in the absence of a catalyst was conducted using the same steps as above. Analysis with a UV-Vis spectrometer showed no degradation of the dye over time in the absence of the catalyst.

## 3. Results and Discussion

### 3.1. X-ray Diffraction (XRD)

**Figure 1:**
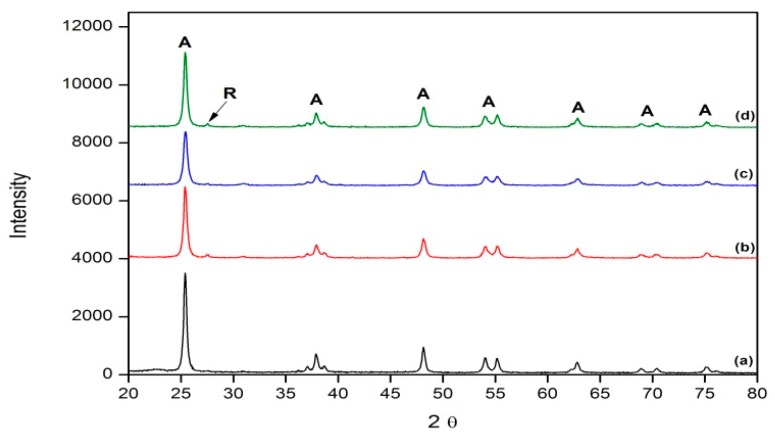
Comparison of X-ray diffraction (XRD) analyses of graphitic carbon nitride (*g*-C_3_N_4_)-doped TiO_2_ samples at varying doping ratios calcined at 600 °C. (**a**) undoped TiO_2_; (**b**) 2%; (**c**) 4%; and (**d**) 8%.

XRD analysis was carried out on each TiO_2_ sample in order to determine their phase compositions when doped with *g*-C_3_N_4_ (Figure S1). A typical XRD diffraction pattern of *g*-C_3_N_4_ consists of two characteristic peaks located at 13.3° and 27.5°, similar to those stated in literature (13.1° and 27.7° respectively [17]). From these results, a clear anatase phase was identified in all samples with similar particle sizes (≈22 nm (Table 1)), calculated using the Scherrer equation (Equation (1)). A slight rutile phase is present in each sample (Figure 1), implying that this method of doping TiO_2_ does not inhibit the anatase to rutile transition (Table 2).

### 3.2. Raman Spectroscopy

**Figure 2:**
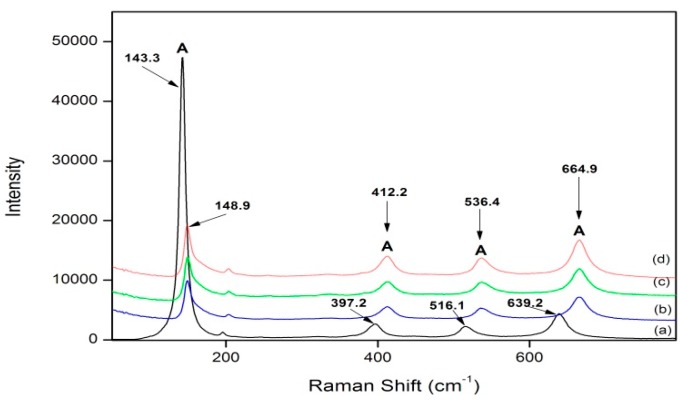
Comparison of Raman analyses of *g*-C_3_N_4_-doped TiO_2_ samples at varying doping ratios calcined at 600 °C. (**a**) undoped TiO_2_; (**b**) 2%; (**c**) 4%; and (**d**) 8%. A = Anatase.

Raman spectroscopy was used to analyze the samples and to identify their phase, whether it be anatase or rutile, along with any potential shifts in the characteristic TiO_2_ peaks. From analysis of the samples calcined at 600 °C, all samples were in the anatase phase. When comparing the undoped TiO_2_ to the doped materials, a substantial up-field shift is observed upon the addition of *g*-C_3_N_4_ (Figure 2). An up-field shift is indicative of the formation of new bonds or bond modifications; therefore, it can be said that *g*-C_3_N_4_ could in fact be doping into the TiO_2_ system rather than existing purely as a mixture of the two.

### 3.3. Fourier Transform Infrared Spectroscopy (FT-IR)

**Figure 3:**
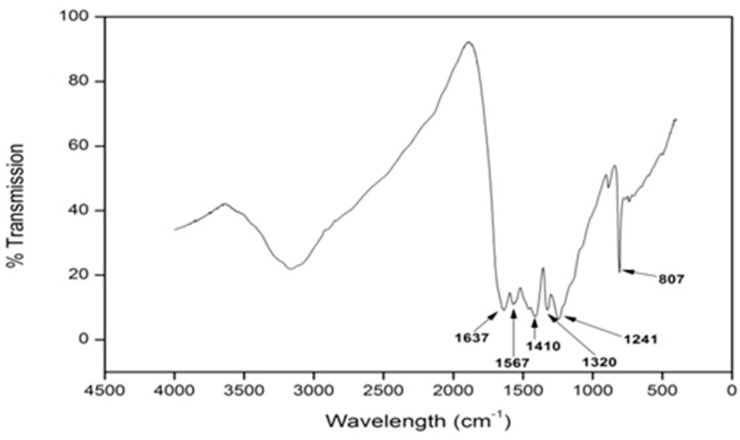
Fourier Transform Infrared (FT-IR) spectra of *g*-C_3_N_4_.

**Figure 4:**
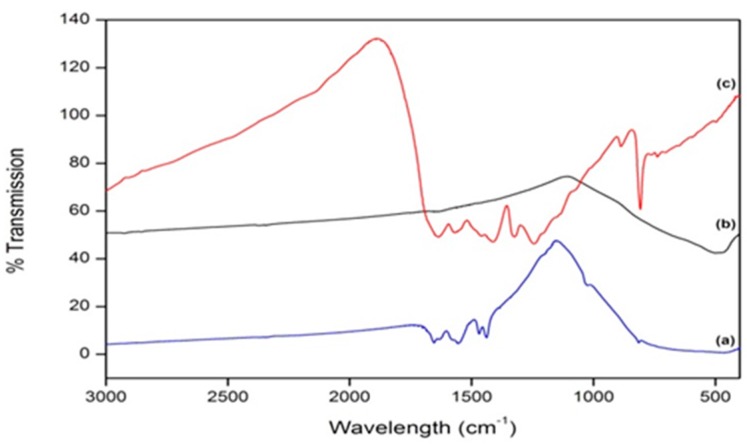
FT-IR spectra comparing (**a**) 4% *g*-C_3_N_4_–TiO_2_; (**b**) undoped TiO_2_; and (**c**) *g*-C_3_N_4_.

With the use of FT-IR, *g*-C_3_N_4_ was easily identified. Peaks associated with *g*-C_3_N_4_ are generally located in the region of 800–1700 cm^−1^, with the characteristic peaks highlighted in Figure 3. The peaks in the region of 1200–1650 cm^−1^ are representative of the absorption peaks of *g*-C_3_N_4_, namely, the stretching and rotation vibration of C-N and C=N bonds [10,14]. The broad peak that appears in the region of 2900–3600 cm^−1^ is indicative of the stretching modes of the terminal NH_2_ or NH groups at the defect sites of the aromatic ring [10,14]. A sharp peak detected at 807 cm^−1^ is due to the characteristic breathing mode of the triazine group [9,14]. A comparison between *g*-C_3_N_4_, undoped TiO_2_, and samples doped at a level of 4% *g*-C_3_N_4_–TiO_2_ is highlighted in Figure 4. From these results, it is clear that the *g*-C_3_N_4_ is present within the system, but it remains unclear from these results whether it is present as a mixture of the two or whether it has successfully doped into the TiO_2_ lattice to some extent.

### 3.4. X-ray Photoelectron Spectroscopy (XPS)

**Figure 5:**
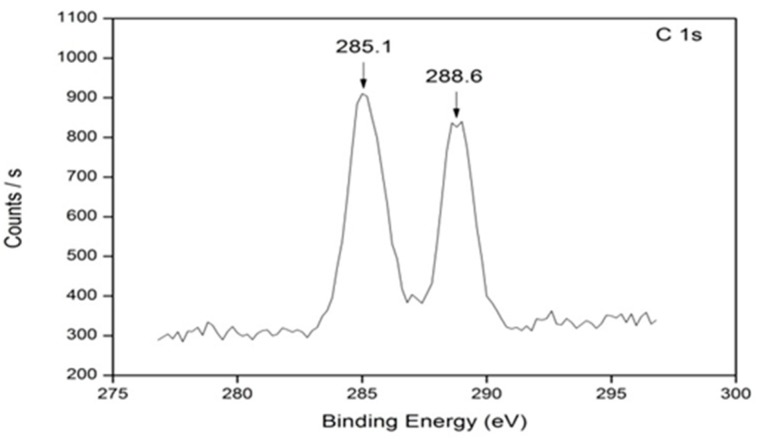
X-ray photoelectron spectroscopy (XPS) spectra of the C 1s peak for sample 4% *g*-C_3_N_4_–TiO_2_.

Analysis of all samples doped with various ratios of *g*-C_3_N_4_ was conducted to help elucidate the possible interactions occurring within the system. All samples showed the presence of four different species; C 1s, O 1s, Ti 2p, and N 1s (Figure S2). The amount of Ti present is identical for each sample at approximately 23% (Table 3). The Ti 2p peak at ≈ 458.6 eV is indicative of the Ti present in its tetravalent state (titanium dioxide), while the absence of a Ti peak at 457.4 eV suggests that surface oxygen vacancies are not forming through this doping method. Nitrogen was present in all samples, including the undoped sample, even though this amount was negligible (0.24 atom %). Two different N 1s positions can be clearly identified: the presence of nitrogen at 401.8 eV (undoped TiO_2_) and 399.8 eV (doped samples). The occurrence of nitrogen at 401.8 eV may be a result of surface contamination. A peak shift to 399.7 eV suggests the presence of lattice bonding in the form of O-Ti-N, where an oxygen atom is replaced by a nitrogen atom. A carbon peak present at ≈ 285 eV is due to simple C-C/C-H bonding within the system. In the doped samples, a second carbon peak appears at 288.6 eV (Figure 5). This peak is attributed to the sp^2^- hybridized carbon in an aromatic ring attached to three nitrogen atoms [43,44], such as the bonding in *g*-C_3_N_4_. A large shift in the oxygen peak is observed upon the doping of TiO_2_ with *g*-C_3_N_4_. In the undoped TiO_2_ samples, a peak at 534.0 eV shows the presence of carbon functional groups with the oxygen. The oxygen peak is shifted toward a lower binding energy upon the addition of *g*-C_3_N_4_, and is measured at 529.8 eV. This new peak shows the bonding between oxygen and a metal—in this case, the bonding between titanium and oxygen in the form of Ti-O. A low atomic concentration for nitrogen was identified in all samples, leading us to believe that the nitrogen is being burnt off from the samples before or at this temperature.

### 3.5. Diffuse Absorbance

With *g*-C_3_N_4_ possessing a band gap of 2.7 eV (460 nm) [6,20], it was important to consider how this low band gap would influence that of the TiO_2_ (3.2 eV). The effect of *g*-C_3_N_4_ doping on the optical properties of these prepared materials was measured using UV-Vis spectroscopy (Figure S3). From the results (Table 4), a low band gap of 2.82 eV was calculated for pure *g*-C_3_N_4_. The value measured for undoped TiO_2_ (600 °C) was 3.15 eV, also similar to that stated in the literature (3.2 eV) [45]. Upon the addition of the dopant to TiO_2_, a shift to higher wavelengths was observed. The presence of rutile in TiO_2_ generally dominates the band gap value; the material present with the lowest band gap will be the value returned. Yet this is usually only observed when rutile is present at a significantly high concentration to result in an effect. In these samples, the majority phase present is anatase with the maximum value of rutile present at 10.2% (4% *g*-C_3_N_4_–TiO_2_); therefore, any reduction in the band gap, however slight it is, may be attributed to doping of TiO_2_ by the dopant, *g*-C_3_N_4_.

### 3.6. Photoluminescence (PL)

**Figure 6:**
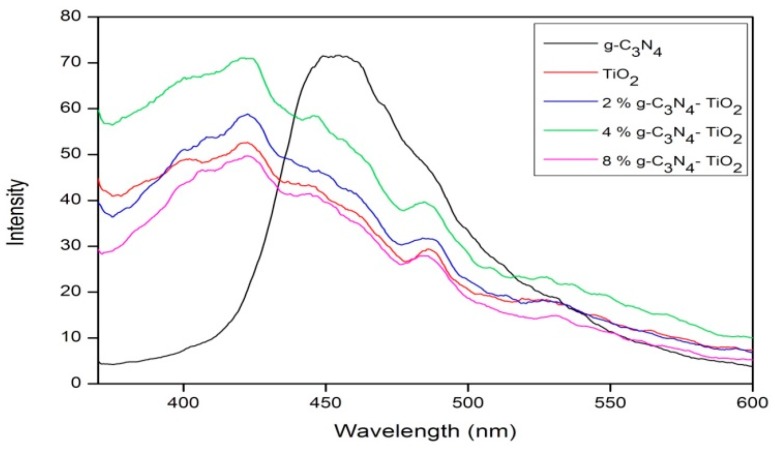
Photoluminescence (PL) spectra of *g*-C_3_N_4_, undoped TiO_2_, 2% *g*-C_3_N_4_–TiO_2_, 4% *g*-C_3_N_4_–TiO_2_, and 8% *g*-C_3_N_4_–TiO_2_.

Photoluminescence (PL) analysis was conducted in order to determine the electron-hole recombination properties of the doped samples. Pure *g*-C_3_N_4_ exhibited a strong, broad peak centered at approximately 455 nm (Figure 6), which can be assigned to the band–band PL phenomenon with the energy of light approximately equal to the band gap energy of *g*-C_3_N_4_ [14,23]. Undoped TiO_2_ also displays a broad peak, with a maximum at ≈ 425 nm. Undoped TiO_2_ has a lower maximum peak than that of *g*-C_3_N_4_, indicating that the TiO_2_ has a slower recombination rate, explained by the larger band gap of TiO_2_ compared to that of *g*-C_3_N_4_. When the dopant and TiO_2_ were mixed at various ratios, an increase in the peak intensity was detected for all samples, except 8% *g*-C_3_N_4_–TiO_2_, which showed a slight decrease. A decrease is explained by a reduced electron-hole recombination rate, yet the increase observed in the 2% *g*-C_3_N_4_–TiO_2_ and 4% *g*-C_3_N_4_–TiO_2_ samples highlights the ineffectiveness of this doping system at reducing the recombination rate [23].

### 3.7. Photocatalysis

**Figure 7:**
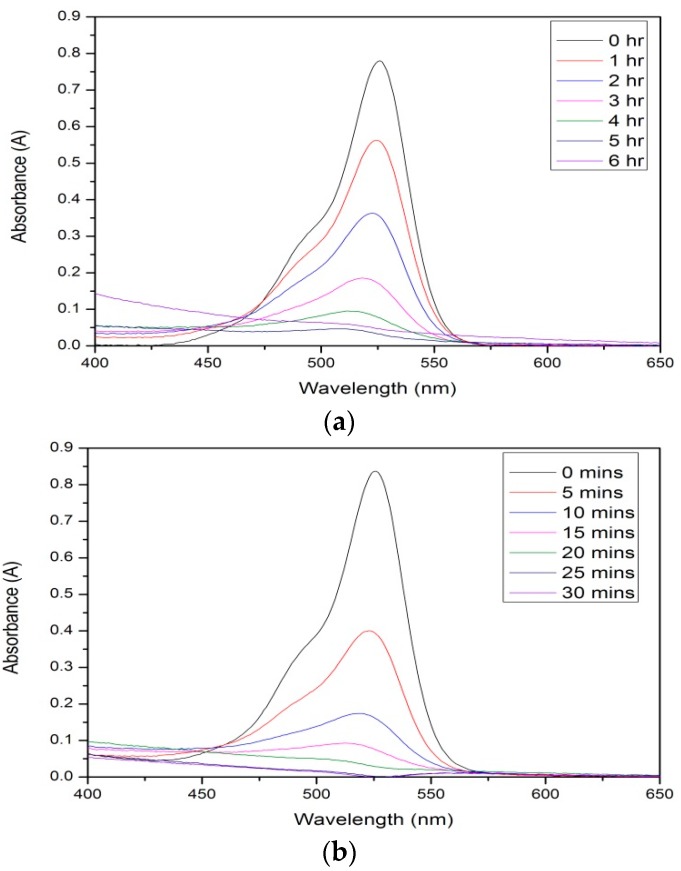
Absorption spectra of rhodamine dye degradation by sample 4% *g*-C_3_N_4_–TiO_2_, 600 °C under (**a**) visible light and (**b**) UV light irradiation.

Photocatalytic studies were carried out in order to measure the decomposition of rhodamine 6G dye under visible light irradiation and UV light irradiation in order to determine the rates of reaction for each sample using first-order rate kinetics. These results were also compared to the measured results for undoped TiO_2_ and the dopant, *g*-C_3_N_4_, individually (Table 5, Table S1). From the obtained results, pure *g*-C_3_N_4_ showed little to no activity under visible light (0.002 min^−1^) and UV light irradiation (0.034 min^−1^). Pure TiO_2_ exhibited much higher rates of degradation in comparison; measurements for visible light and UV light were 0.012 min^−1^ and 0.130 min^−1^, respectively. When the mixed samples were analyzed under visible light, little activity was measured for all doped samples—results similar to that of pure TiO_2_. This result indicates that the samples are not visible light active materials, which coincides with the band gap calculations showing band gaps similar to that of anatase (≈3.1 eV). When the light source was changed to a UV lamp, all doped samples showed high activity. The samples prepared with 2% *g*-C_3_N_4_ had an increased rate of reaction over TiO_2_ (0.141 min^−1^), with the rate increasing yet again when the loading was increased to 4% (0.187 min^−1^) (Figure 7). Upon further addition of *g*-C_3_N_4_ to 8%, a rate decrease was observed to a level below that of TiO_2_ (0.103 min^−1^). The inactivity of the pure *g*-C_3_N_4_ could be in fact hindering the photocatalytic rate of activity at this higher doping level due to surface poisoning.

## 4. Discussion

A simple method for the preparation of *g*-C_3_N_4_ with subsequent doping into TiO_2_ was studied. It was the hope to develop a material which exhibited an effective charge separation between *g*-C_3_N_4_ and TiO_2_. Effective charge separation exists when the photogenerated holes in TiO_2_ are transferred from the TiO_2_ valence band to the highest occupied molecular orbital (HOMO) of *g*-C_3_N_4_ with additional injection of the electrons from the lowest unoccupied molecular orbital (LUMO) of *g*-C_3_N_4_ to the conduction band of TiO_2_ [14].

From XRD analysis, it can clearly be observed that the *g*-C_3_N_4_ has been synthesized through the thermal decomposition of melamine with characteristic peaks located at 13.3° and 27.5°, similar to those stated in the literature (Figure 1). When this material was added to TiO_2_, the particle sizes calculated were similar to that of pure TiO_2_, showing that the dopant did not affect the resulting particle sizes. As stated previously, the anatase to rutile (ART) transition generally occurs at around 600–700 °C [46,47,48]. Using the Spurr equation (Equation (2)), it was calculated that undoped TiO_2_ retained 100% anatase at 700 °C, with 5% rutile formed at 800 °C. Table 1 shows that upon the addition of *g*-C_3_N_4_, the development of rutile is promoted and is present in small values (10% rutile at 4% *g*-C_3_N_4_–TiO_2_). This shows that this method of doping TiO_2_ does not inhibit the ART temperature, but reduces the temperature to lower than that of the undoped TiO_2_. Rather than achieving high temperature stable anatase, it was hoped that the as-prepared materials could be effective as photocatalysts for processes which require low temperatures.

To confirm the formation of *g*-C_3_N_4_, techniques such as FT-IR spectroscopy and XPS analysis were employed. FT-IR clearly showed the formation of the *g*-C_3_N_4_ (Figure 3) and a comparison between undoped TiO_2_, *g*-C_3_N_4_, and 4% *g*-C_3_N_4_–TiO_2_ showed the presence of the dopant alongside the TiO_2_ phase. XPS analysis confirmed the presence of the *g*-C_3_N_4_ with a carbon peak at approximately 288.6 eV (Figure 5). Effective doping of nitrogen into the titania lattice, with a shift in the 401.8 eV peak (undoped) to 399.7 eV (doped), was witnessed in all doped samples. This is due to the formation of the O–Ti–N bond.

To determine whether the *g*-C_3_N_4_ was effectively doped into the TiO_2_ lattice and is not purely present in a mixture with TiO_2_, Raman spectroscopy was utilized. Apart from being a complimentary technique to determine phase identification, Raman spectroscopy can highlight changes in the TiO_2_ system associated with phase change, bond modifications, and the formation of new bonds. By comparing undoped TiO_2_ to *g*-C_3_N_4_-doped TiO_2_, a large progressive shift towards higher wavenumbers occurs for all characteristic anatase peaks. For example, when undoped and 8% *g*-C_3_N_4_–TiO_2_ were compared, the following shifts were noted: 143.6 to 148.9 cm^−1^, 397.2 to 412.2 cm^−1^, 516.1 to 536.4 cm^−1^, and 639.2 to 664.9 cm^−1^. Shifting to a higher wavenumber in Raman spectroscopy is associated with phase change, bond modifications, and the formation of new bonds. This result is reflected in the XPS data (Table 3), where nitrogen bonding through the replacement of an oxygen atom, O–Ti–N, is detected only after the doping process.

When the XPS results for oxygen and titania were analyzed, it was confirmed that both surface hydroxyl groups (O 1s peak at 531.4 eV) and surface oxygen vacancies (Ti 2p peak at 457.4 eV) were absent. The absent Ti 2p peak at 457.4 eV indicates that the Ti^4+^ is not being reduced to Ti^3+^. The reduction of Ti^4+^ to Ti^3+^ can occur through two main processes. The first is when a photoelectron, generally generated through light irradiation equal to or greater than the band gap energy of TiO_2_, is trapped on the surface leading to the reduction of the Ti^4+^ cation to the Ti^3+^ state [49]. The second process occurs when there is a loss of oxygen from the TiO_2_ surface when exposed to a reducing atmosphere, such as H_2_ or CO, during the thermal treatment process [49]. Using carbon as a dopant has been proven to be an efficient method of producing these surface oxygen vacancies (Ti^3+^ states) [50,51]. Xiao-Quan *et al.* [50] showed that, through the pyrolysis of titanyl organic compounds, sufficient carbon was formed to induce the reduction of Ti^4+^ to Ti^3+^ along with the production of surface hydroxyl radicals. It is these two forms that contribute significantly to the overall photocatalytic ability of TiO_2_ by inhibiting the recombination process and even allowing for increased visible light activity [52,53,54,55,56]. Without these surface defects and hydroxyl groups, it can be said that the dopant, *g*-C_3_N_4_, will not improve the overall activity of the photocatalyst.

To assess the photoactivity of the doped materials, photocatalysis studies on the degradation of rhodamine 6G dye were conducted under both UV and visible light irradiation. Interesting results were obtained throughout this study, with 8% *g*-C_3_N_4_–TiO_2_ proving to have the lowest rate of reactivity under UV light irradiation (0.103 min^−1^), and exhibiting little or no reaction when irradiated with a visible light source (0.009 min^−1^). When BET was utilized to determine the surface area of the 4% and 8% *g*-C_3_N_4_–TiO_2_ samples, very similar values were obtained—29.4 and 28.5 m^2^/g—suggesting that the actual surface area is not a factor. This could be due to an overloading of the system, with the covering of the active sites resulting in a lower reaction rate. Perhaps a loading of 8% *g*-C_3_N_4_ is too much, and some active sites on the TiO_2_ surface are being covered and unable to take part during the photocatalytic reactions. The best sample during the photocatalytic study was 4% *g*-C_3_N_4_–TiO_2_ with a rate of reaction calculated to be 0.187 min^−1^ under UV light irradiation. Compared to the undoped TiO_2_ under this light source (0.130 min^−1^), this sample was 1.4 times faster.

## 5. Conclusions

The use of *g*-C_3_N_4_ has been previously reported to be effective in the reduction in recombination through the interaction between the two interfaces of TiO_2_ and *g*-C_3_N_4_. It can be confirmed that a composite of the materials was formed (Raman) and not just a mixture of the two. The composite systems show an alteration of the band gap energies for all doped samples when compared to the two materials alone, leading us to believe that nitrogen, detected by XPS, could in fact play a role in altering the band gap. In terms of the photocatalytic ability of the doped samples, an increased reactivity was measured for the 2% and 4% *g*-C_3_N_4_–TiO_2_ samples with a reduction in the reaction rate for the 8% *g*-C_3_N_4_–TiO_2_ sample observed under UV light irradiation. Since we have identified by XPS analysis that surface hydroxyl radicals and oxygen vacancies are not being formed throughout this preparation, it can be suggested that the increased photocatalytic reaction rates are due to successful interfacial interactions with the *g*-C_3_N_4_-doped TiO_2_ systems.

## Figures and Tables

**Table 1:** Particle size (nm) estimations of *g*-C_3_N_4_-doped TiO_2_ samples at varying doping ratios calcined at 600 °C calculated using the Scherrer equation.

Sample Name	Particle Size (nm)	
	A	R
Blank, TiO_2_	23.9	0
2% *g*-C_3_N_4_–TiO_2_	21.7	28.5
4% *g*-C_3_N_4_–TiO_2_	22.7	46.8
8% *g*-C_3_N_4_–TiO_2_	22.2	36.8

**Table 2:** Anatase/rutile percentage of *g*-C_3_N_4_-doped TiO_2_ samples at varying doping ratios calcined at 600 °C.

Sample Name	Anatase Percentage (%)	Rutile Percentage (%)
Blank, TiO_2_	100	0
2% *g*-C_3_N_4_–TiO_2_	93.3	6.7
4% *g*-C_3_N_4_–TiO_2_	89.8	10.2
8% *g*-C_3_N_4_–TiO_2_	92.7	7.3

**Table 3:** XPS analysis of *g*-C_3_N_4_-doped TiO_2_ samples at varying doping ratios calcined at 600 °C.

	Temperature (°C)	C 1s	C 1s	O 1s	Ti 2p	N 1s
Binding energy (eV)						
TiO_2_	600	284.9	–	534.0	458.5	401.8
2%	600	285.0	288.6	529.8	458.6	399.8
4%	600	285.1	288.6	529.9	458.7	399.8
8%	600	285.1	288.6	529.9	458.7	399.4
Atomic %						
TiO_2_	600	10.6		65.2	23.9	0.2
2%	600	11.9 *		64.9	22.9	0.3
4%	600	11.7 *		64.8	23.4	0.2
8%	600	11.6*		65.1	23.0	0.3

* denotes the total carbon content (atom %).

**Table 4:** Calculated band gap values for *g*-C_3_N_4_-doped TiO_2_ samples.

Sample	Temperature (°C)	Band Gap (eV) (± 0.1 eV)	
		Absorption Edge (nm)	Band Gap (eV)
*g*-C_3_N_4_	600	439.8	2.82
TiO_2_	600	393.7	3.15
2% *g*-C_3_N_4_–TiO_2_	600	404.8	3.06
4% *g*-C_3_N_4_–TiO_2_	600	400.8	3.09
8% *g*-C_3_N_4_–TiO_2_	600	398.7	3.11

**Table 5:** Rates of reactions for Rhodamine 6G degradation under UV light irradiation.

Sample Name	Temperature (°C)	*k* (min^−1^)	
		UV	Visible
Undoped TiO_2_	600	0.130	0.012
*g*-C_3_N_4_	600	0.034	0.005
2% *g*-C_3_N_4_–TiO_2_	600	0.141	0.009
4% *g*-C_3_N_4_–TiO_2_	600	0.187	0.009
8% *g*-C_3_N_4_–TiO_2_	600	0.103	0.011

## Supplementary Materials

The following are available online at www.mdpi.com/1996-1944/9/4/286/s1.
