# Supplementary material for: Photocatalytic Properties of g-C3N4–TiO2 Heterojunctions under UV and Visible Light Conditions
Source: Materials (Basel). 2016 Apr 14;9(4):286. doi: 10.3390/ma9040286 (PMC5502979; doi:10.3390/ma9040286)
Supplement: Supplementary file 1 [file materials-09-00286-s001.pdf]

Supplementary Materials: Photocatalytic Properties of *g*-C_3N_4–TiO_2 Heterojunctions under UV and Visible Light Conditions

Rachel Fagan, Declan E. McCormack, Steven J. Hinder and Suresh C. Pillai

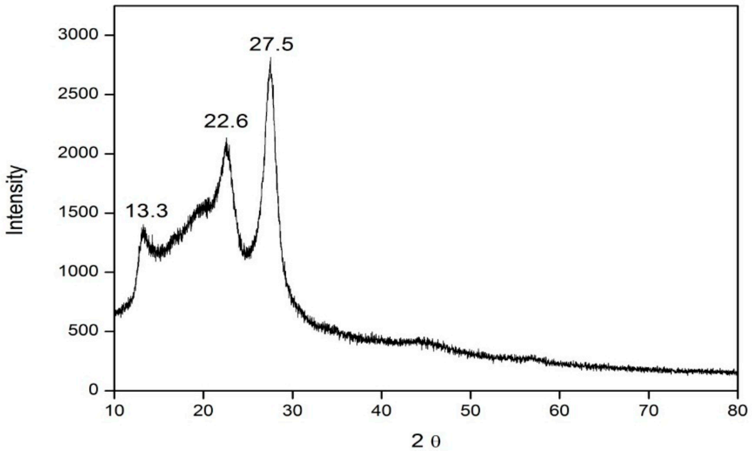

Figure S1. XRD diffraction spectra of *g*-C_3N_4.

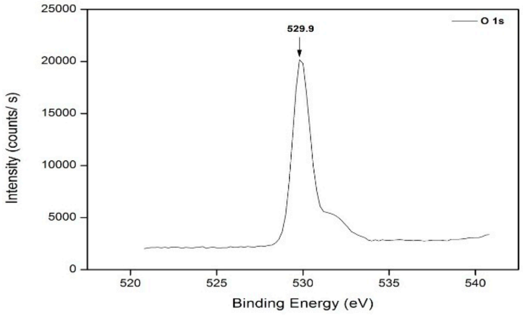

(**a**)

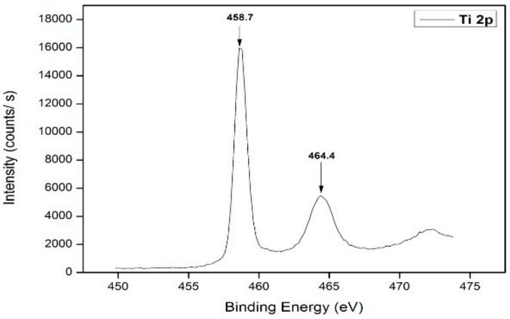

(**b**)

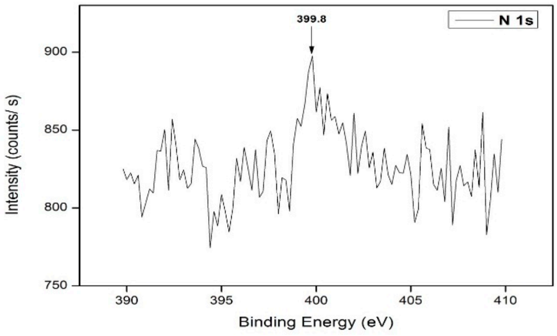

(**c**)

Figure S2. XPS spectra of 4% *g*-C_3N_4/TiO_2 calcined at 600 °C. (**a**) O 1s; (**b**) Ti 2p and (**c**) N 1s.

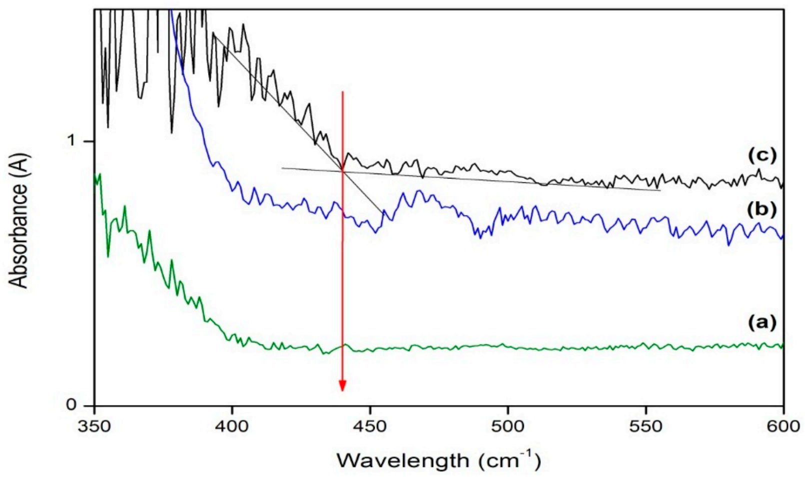

Figure S3. Comparison of diffuse absorbance spectra: (**a**) TiO_2 (600 °C); (**b**) 4% *g*-C_3N_4/TiO_2 (600 °C) and (**c**) *g*-C_3N_4.

Table S1. Standard deviation values.

Sample Name	Temp (˚C)	Rate of Reaction (min^{-1}) Average Values Standard Deviation	
		Visible	UV
TiO_2	600	0.0005	0.0309
g-C_3N_4	600	0.0006	0.0092
2% *g*-C_3N_4/TiO_2	600	0.0013	0.0124
4% *g*-C_3N_4/TiO_2	600	0.0004	0.0583
8% *g*-C_3N_4/TiO_2	600	0.0011	0.0006
